# High external pH enables more efficient secretion of alkaline α-amylase AmyK38 by *Bacillus subtilis*

**DOI:** 10.1186/1475-2859-11-74

**Published:** 2012-06-08

**Authors:** Kenji Manabe, Yasushi Kageyama, Masatoshi Tohata, Katsutoshi Ara, Katsuya Ozaki, Naotake Ogasawara

**Affiliations:** 1 Biological Science Laboratories, Kao Corporation, 2606 Akabane Ichikai, Haga, Tochigi, 321-3497, Japan; 2 Graduate School of Information Science, Nara Institute of Science and Technology, 8916-5 Takayama, Ikoma, Nara, 630-0101, Japan

**Keywords:** α-Amylase, AmyK38, *Bacillus subtilis*, CssRS, Genome reduction, HtrA, HtrB, MGB874, Secretion stress response

## Abstract

****Background**:**

*Bacillus subtilis* genome-reduced strain MGB874 exhibits enhanced production of exogenous extracellular alkaline cellulase Egl-237 and subtilisin-like alkaline protease M-protease. Here, we investigated the suitability of strain MGB874 for the production of α-amylase, which was anticipated to provoke secretion stress responses involving the CssRS (Control secretion stress Regulator and Sensor) system.

****Results**:**

Compared to wild-type strain 168, the production of a novel alkaline α-amylase, AmyK38, was severely decreased in strain MGB874 and higher secretion stress responses were also induced. Genetic analyses revealed that these phenomena were attributable to the decreased pH of growth medium as a result of the lowered expression of *rocG*, encoding glutamate dehydrogenase, whose activity leads to NH_3_ production. Notably, in both the genome-reduced and wild-type strains, an up-shift of the external pH by the addition of an alkaline solution improved AmyK38 production, which was associated with alleviation of the secretion stress response. These results suggest that the optimal external pH for the secretion of AmyK38 is higher than the typical external pH of growth medium used to culture *B. subtilis*. Under controlled pH conditions, the highest production level (1.08 g l^-1^) of AmyK38 was obtained using strain MGB874.

****Conclusions**:**

We demonstrated for the first time that RocG is an important factor for secretory enzyme production in *B. subtilis* through its role in preventing acidification of the growth medium. As expected, a higher external pH enabled a more efficient secretion of the alkaline α-amylase AmyK38 in *B. subtilis*. Under controlled pH conditions, the reduced-genome strain MGB874 was demonstrated to be a beneficial host for the production of AmyK38.

## **Background**

*Bacillus subtilis* is a Gram-positive sporiferous bacillus with numerous attractive characteristics for the production of industrially important enzymes, including high growth rate, protein secretion ability, and Generally Regarded as Safe (GRAS) status [1-4]. *B. subtilis* has also been extensively characterized by biochemical, genetic, and molecular biological studies, and the complete sequencing of the *B. subtilis* strain 168 genome has facilitated genetic engineering of this model organism [5,6]. Recently, we constructed a series of multiple-deletion mutant strains by the sequential deletion of 865 genes (874 kb; 20.7% of total genomic DNA), including all prophage and prophage-like sequences, the *pks* and *pps* operons, and 11 non-essential gene clusters, from *B. subtilis* strain 168 [7,8]. One of the generated mutants, strain MGB874, showed enhanced production of exogenous extracellular alkaline cellulase Egl-237 [9] and a subtilisin-like alkaline protease M-protease [10], which were expressed from a multi-copy plasmid. We demonstrated that two factors contribute to the high enzyme production in reduced-genome strain MGB874: increased specific productivity and improved cell yields [11]. The former is likely attributable to higher target gene expression due to increased promoter activity and plasmid copy number, while the latter may result from an increased supply of glutamate by enhanced glutamate metabolism due to deletion of the *rocDEF-rocR* region. Therefore, strain MGB874 would be a suitable host for the high-level production of other beneficial industrial enzymes.

α-Amylase (α-1,4-glucan-4-glucanohydrolase, EC3.2.1.1) is an important enzyme in the food and detergent industries. We previously isolated a novel α-amylase, AmyK38 (55,097 Da), from alkaliphilic *Bacillus* sp. strain KSM-K38 and demonstrated this enzyme has several unique properties, including high activity at alkaline pH and strong resistance to chemical oxidants [12]. In addition, although α-amylases are generally metalloenzymes that contain at least one activating and stabilizing Ca^2+^ ion [13], AmyK38 does not associate with Ca^2+^ ions; rather, its enzyme activity depends on Na^+^ ions and is not inhibited by chelating reagents. Due to these characteristics, AmyK38 would likely be an advantageous component of laundry and dishwashing detergents, which are alkaline solutions and typically contain bleach and chelating agents. However, the improvement of AmyK38 production levels is a necessary prerequisite for the commercial use of this α-amylase, which is only produced by strain KSM-K38 at markedly low levels (30 mg l^-1^) [12].

In *B. subtilis*, the overproduction of α-amylases from *Bacillus licheniformis* (AmyL) and *Bacillus amyloliquefaciens* (AmyQ) provokes a CssRS (Control secretion stress Regulator and Sensor) dependent secretion stress response [14,15], which represents a significant bottleneck for high-level enzyme production [16,17]. The misfolded or aggregated forms of AmyL and AmyQ at the membrane-cell wall interface is thought to be detected by the membrane sensor protein CssS [15], which then activates the response regulator CssR through phosphorylation. Consequently, activated CssR leads to increased transcription of *htrA* and *htrB*, which encode putative membrane-bound proteases [14]. Previous studies have shown that inactivation of the *dlt* operon, which is involved in the D-alanylation of cell wall teichoic acids, leads to the stabilization and increased production of AmyQ in *B. subtilis*, likely due to increased negative charge density in the cell wall [18]. Furthermore, the inactivation of *dlt* operon led to a strongly reduced AmyQ-induced secretion stress response as compared to the wild-type strain [19].

Hagihara et al. [20] reported that the amino acid sequence of AmyK38 exhibits moderate overall identity with AmyL (62.8%) and AmyQ (59.5%). However, it is not clear whether the overproduction of AmyK38 provokes a secretion stress response in *B. subtilis*. Here, we investigated the production and secretion stress levels of AmyK38 in genome-reduced strain MGB874.

## **Results and discussion**

### **Genome reduction significantly decreases production of AmyK38**

**Figure 1:**
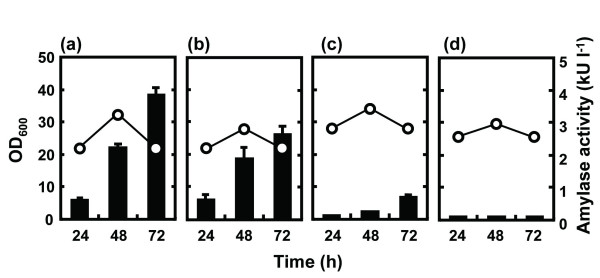
**AmyK38 production by multiple-deletion strains of*B. subtilis.****B. subtilis* strains 168 (**a**), MGB625 (**b**), MGB723 (**c**), and MGB874 (**d**) harboring pHYK38 were cultured in 2xL-Mal medium with shaking at 30°C. Culture OD_600_ (open circles) and α-amylase activity in the growth medium (black bars) were evaluated. Strain 168 is a wild-type strain and strains MGB625, MGB723, and MGB874 are multiple-deletion mutants. The size of the genomic deletions increases in the order MGB625, MGB723, and MGB874. The results presented are the means of three individual experiments. Error bars represent standard deviations (n = 3).

We first evaluated the production levels of exogenous AmyK38 in wild-type strain 168 and a series of multiple-deletion mutants using plasmid pHYK38 for the overexpression of AmyK38 protein. After 72 h cultivation in 2xL-Mal medium, the α-amylase activity in the culture broth of each strain was measured (Figure 1). As α-amylase activity was barely detected in the culture supernatant of *B. subtilis* harboring pHY300PLK (empty vector), the indirect effects of endogenous α-amylase production were considered to be negligible under the assay conditions used in this study. In contrast to our previous report on the enzymes cellulase Egl-237 and M-protease [8], we found that the production of AmyK38 gradually decreased in the order of strains 168, MGB625, MGB723, and MGB874, and was markedly impaired as a result of the genomic deletion process between strains MGB625 and MGB723 (Figure 1). As no significant differences in culture optical density (OD_600_) or cell viabilities were detected (data not shown), the decrease in AmyK38 production was not caused by impaired cell growth. Based on the measured α-amylase activity (Figure 1), it was estimated that the amount of AmyK38 produced in strains 168, MGB625, MGB723, and MGB874 after 72 h of culture was 1.68 x 10^2^, 1.15 x 10^2^, 30.0, and 4.57 mg l^-1^, respectively.

### **Investigation of the bottleneck in AmyK38 production in genome-reduced strains**

**Figure 2:**
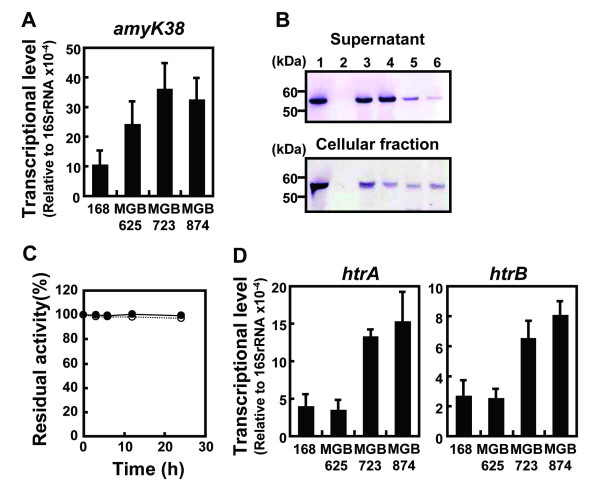
**Investigation of the cause of decreased AmyK38 production.***B subtilis* strains 168 (**a**), MGB625 (**b**), MGB723 (**c**), and MGB874 (**d**) harboring pHYK38 were cultured in 2xL-Mal medium with shaking at 30°C. (**A**) Transcriptional levels of *amyK38* were determined by qRT-PCR after 48 h of cultivation and are reported relative to those of 16 S rRNA. (**B**) Secretion and cellular amounts of AmyK38 in the genome-reduced strains. The concentrations of AmyK38 in supernatants and cellular fractions after 48 h of cultivation were analyzed by Western blotting. Supernatants were diluted 40-fold and 3 μl was used for analysis. Cells were re-suspended to an OD_600_ of 3.0, corresponding to an approximately 10-fold dilution of cultures, 12 μl samples were used for analysis. Thus, cellular fractions were 16-fold enriched compared to supernatants. The positions of the protein standards are indicated in kDa to the left of the gel. Lanes: 1, purified AmyK38 (10 ng); 2, strain 168 harboring pHY300PLK (empty vector); 3, strain 168 harboring pHYK38; 4, strain MGB625 harboring pHYK38; 5, strain MGB723 harboring pHYK38; and 6, strain MGB874 harboring pHYK38. (**C**) Stability of AmyK38 in spent culture medium. The spent culture media of strains 168 (open circles) and MGB874 harboring pHYK38 (closed circles) were collected after 48 h of cultivation. Time intervals of α-amylase activities of the spent media were measured at 30°C. (**D**) Transcriptional levels of *htrA* and *htrB* after 48 h of cultivation were determined by qRT-PCR and are reported relative to those of 16 S rRNA. The results presented in (**A**) and (**D**) are the means of three individual experiments. Error bars represent standard deviations (n = 3).

We examined whether the observed decrease in AmyK38 production was due to impaired transcription of *amyK38* by quantitative real-time PCR (qRT-PCR). However, we found that the transcriptional levels of *amyK38* were higher for the multiple-deletion mutant cells than those of wild-type strain 168 cells (Figure 2A). This finding of increased target gene transcription in these genome-reduced strains is consistent with our previous study [11] and does not appear to be related to the nature of the gene product.

We next examined the concentration of AmyK38 in culture supernatants and cell fractions by Western blotting. As shown in Figure 2B, the levels of AmyK38 in the supernatants decreased in the order of strains 168, MGB625, MGB723, and MGB874, which closely correlated with the α-amylase activities measured at 48 h (Figure 1). Additionally, the levels of AmyK38 detected in the cellular fraction in the genome-reduced strains were also lower than that of strain 168 (Figure 2B). As the size of AmyK38 observed in the cellular fraction was equivalent to that of purified (mature) AmyK38, these cell-associated AmyK38 would be translocated mature protein that has not yet been released into the growth medium. The isoelectric point (pI) of AmyK38 is 4.2 [12], and AmyK38 we detected in cellular fraction could not be removed by washing with 5 M LiCl (data not shown), suggesting that it is not bound to cell wall, but bound to cell membrane.

To investigate the possible degradation of secreted AmyK38 in the genome-reduced strains, we evaluated the stability of mature AmyK38 in culture medium following the growth of strains 168 and MGB874 harboring pHYK38 to the stationary phase (at 48 h). As shown in Figure 2C, AmyK38 was stable in the spent culture medium of both strains over an incubation period of 24 h at 30°C. This finding indicates that following the folding of AmyK38 into its native conformation and release from the cells into the growth medium, the enzyme is highly stable.

Finally, we measured the transcriptional levels of *htrA* and *htrB,* which encode membrane-bound proteases that respond to secretion stress in *B. subtilis*[14]. The data shown in Figure 2D indicates that the secretion stress levels resulting from the overproduction of AmyK38 were higher in strains MGB723 and MGB874 than those in strains 168 and MGB625. Westers et al. [21] have demonstrated that the intensity of the secretion stress response in *B. subtilis* correlates with the cellular level of AmyQ. However, in the present study, the concentration of AmyK38 in the cellular fraction of strains MGB723 and MGB874 was inversely related to the levels of secretion stress, although the amount of cell-associated AmyK38 was also reduced in MGB625 without induction of HtrA and HtrB.

These findings raise the possibility that unfolded forms of AmyK38 accumulated after translocation and they were degraded rapidly at the membrane-cell wall interface in strains MGB723 and MGB874. It was reported that the predominant proteases responsible for protein degradation at the membrane-cell wall interface are the WprA-derived cell wall-bound protein CWBP52 [22] and CssRS-dependent membrane bound proteases HtrA and HtrB [15]. However, the gene encoding WprA was deleted during the multiple deletion process used to construct strains MGB723 and MGB874 [7,8]. Thus, the main proteases responsible for the degradation of AmyK38 before its release into the growth medium appear to be HtrA and HtrB. In an attempt to confirm this speculation, we evaluated AmyK38 production in *cssRS* mutants, but no transformants harboring pHYK38 for AmyK38 production could be obtained. This result is consistent with previous reports that found inactivation of *cssRS* causes growth defects under secretion stress conditions and does not improve the yield of heterologous proteins [23].

### **Genetic cause for decreased AmyK38 production**

To determine the underlying genetic cause for the significant decrease in AmyK38 production in the genome-reduced strains, we examined the regions deleted during the generation of strain MGB723 from strain MGB625, which involved deletion of the genomic region from *pdp* to *rocR* (nucleotides 4,049,059 to 4,147,133 in sequence NC_000964.3; NCBI GenBank Database [6]). Deletion of this genomic region significantly increased the production of cellulase Egl-237 and M-protease [7,8], with subsequent analyses revealing that deletion of the *rocDEF-rocR* region was responsible for the enhanced enzyme production [11]. However, here, we found that deletion of the *rocDEF-rocR* region caused a significant decrease in the production level of AmyK38 (Additional file 1: Figure S1). Thus, deletion of the *rocDEF-rocR* region has a negative effect on the production of the α-amylase AmyK38, but leads to increased production of Egl-237 and M-protease [11].

**Figure 3:**
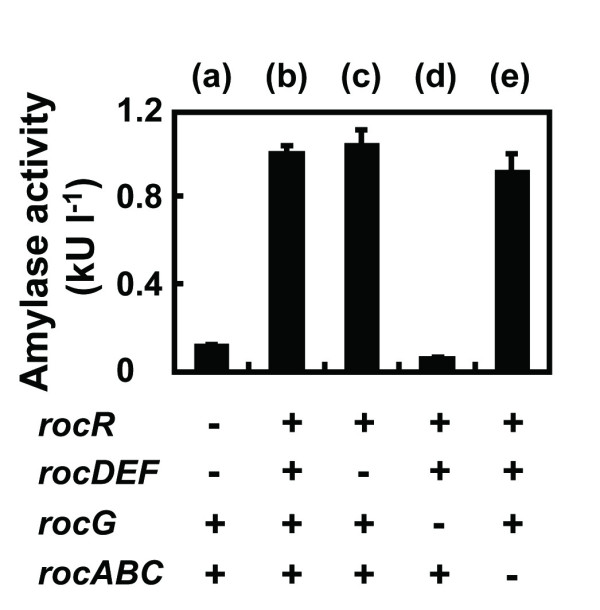
**Contribution of the RocR regulon to amylase production.***B. subtilis* MGB874 derivative strains in the presence (+) or absence (-) of the indicated genes were transformed with pHYK38, and then cultured in 2xL-Mal medium with shaking at 30°C. α-amylase activities were measured after 72 h cultivation. The following strains were evaluated: (**a**), MGB874; (**b**), 874DEFR; (**c**), 874RocR; (**d**), 874DEFRΔrocG; and (**e**), 874DEFRΔrocABC.

RocR is a transcriptional activator of the arginine degradation pathway, which is encoded by the *rocABC* and *rocDEF* operons, and *rocG* gene [24]. To determine the specific gene(s) responsible for the decreased production of AmyK38, we evaluated single-deletion mutants of the RocR regulon (Figure 3). From this analysis, we found that inactivation of the *rocG* gene significantly reduces the production of AmyK38 in *B. subtilis* (Figure 3).

**Figure 4:**
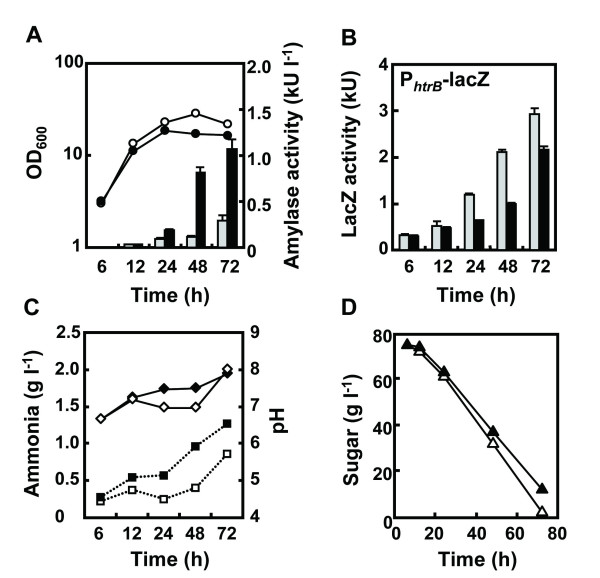
**Effect of*rocG*expression on AmyK38 production, secretion stress responses, and external pH.** Strain R-1926 (MGB874 P_spac_-*rocG* and P_htrB_-*lacZ*) harboring pHYK38 was cultured in 2xL-Mal medium with shaking at 30°C in the absence (white symbols and gray bars) or presence (black symbols and black bars) of 1 mM IPTG. (**A**) Culture OD_600_ (circles) and α-amylase activities in the growth medium (bars). (**B**) Secretion stress levels were determined by measuring β-galactosidase activities of cells expressing a transcriptional *htrB-lacZ* fusion. (**C**) Ammonia concentrations (squares) and pH of culture broth (diamonds). (**D**) Concentrations of sugar in the growth media. All results presented are the averages of three individual experiments. Error bars represent standard deviations (n = 3).

To investigate the relationship between *rocG* expression and AmyK38 production and secretion stress responses, an isopropyl-β-D-thiogalactopyranoside (IPTG)-inducible P_spac_*rocG* gene and transcriptional *htrBlacZ* gene fusion were introduced into strain MGB874, generating strain R-1926 (MGB874 P_spac_*rocG* and P_htrB_*lacZ*). The *htrBlacZ* fusion is commonly used as a reporter of secretion stress in *B. subtilis*[14,15]. Detailed time course analyses of strain R-1926 are shown in Figure 4. Notably, the induction of *rocG* expression by 1 mM IPTG increased the production level of AmyK38, although the culture OD_600_ in the stationary phase were decreased (Figure 4A). Furthermore, the secretion stress response was also decreased by the induction of *rocG* expression (Figure 4B).

RocG is a catabolic glutamate dehydrogenase that catalyzes the conversion of glutamate to 2-oxoglutarate and ammonia [25]. As the deamination of glutamate by RocG is a major ammonia-releasing reaction [26], which would lead to increased external pH, we focused on ammonia generation and changes in external pH of the culture medium (Figure 4C). We found that the ammonia concentrations in the growth media and external pH were higher under *rocG-*induced conditions compared to those measured under non-inducing conditions. In the non-inducing conditions, the production of AmyK38 was arrested in the stationary phase (Figure 4A, white bars, 24 to 48 h). During this growth phase, the external pH was lower (Figure 4C) and the secretion stress levels were higher (Figure 4B) compared to those detected under inducing conditions. Together, these data suggest the possibility that a lower pH leads to decreased extracellular production of AmyK38, which is accompanied by increased levels of secretion stress in *B. subtilis*. At 72 h of cultivation, the external pH for strain MGB874 was drastically increased, likely due to sugar starvation (Figure 4D) [27]. In accordance with this increase in external pH, the production of AmyK38 increased from 48 to 72 h of cultivation under non-inducing conditions of *rocG* (Figure 4A).

### **Up-shift in external pH enhances AmyK38 production levels and decreases secretion stress**

**Figure 5:**
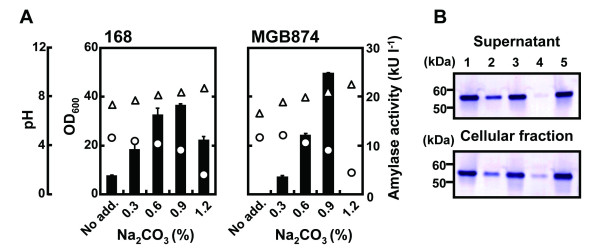
**Effect of pH up-shift on AmyK38 production.** (**A**) *B. subtilis* strains 168 and MGB874 harboring pHYK38 were grown in 2xL-Mal medium at 30°C. At the transition phase (12 h), a sodium carbonate solution (Na_2_CO_3_) was added to the growth medium at the indicated final concentrations (w/v). Culture OD_600_ (open circles) and external pH (open triangles) were measured after 24 h of cultivation, and α-amylase activities (black bars) were measured after 72 h cultivation. All results presented are the averages of three individual experiments. Error bars represent standard deviations (n = 3). (**B**) Secretion and cellular amounts of AmyK38 in strains 168 and MGB874 harboring pHYK38. Cells were grown in 2xL-Mal medium at 30°C until the transition phase (12 h), at which point Na_2_CO_3_ was added to the growth medium at a final concentration of 0.9% (w/v). The amounts of AmyK38 in supernatants and cellular fractions after 48 h of cultivation were analyzed by Western blotting. Supernatants were diluted 240-fold and 3 μl sample volumes were used for analysis. Cells were re-suspended to an OD_600_ of 3.0, corresponding to an approximately 10-fold dilution, and 12 μl sample volumes (cell fraction) were analyzed. The positions of the protein standards are indicated in kDa to the left of the gels. Lanes: 1, purified AmyK38 (10 ng); 2, strain 168 harboring pHYK38 without Na_2_CO_3_ addition; 3, strain 168 harboring pHYK38 with Na_2_CO_3_ addition; 4, strain MGB874 harboring pHYK38 without Na_2_CO_3_ addition; and 5, strain MGB874 harboring pHYK38 with Na_2_CO_3_ addition.

To investigate the effects of external pH on AmyK38 production, we added different concentrations of sodium carbonate (Na_2_CO_3_) as an alkaline solution to the growth medium during the transition phase (12 h of cultivation) (Figure 5A). Surprisingly, the addition of up to 0.9 % (w/v) Na_2_CO_3_ significantly improved α-amylase production not only by strain MGB874, but also by strain 168, and this effect far exceeded the increase resulting from the induction of *rocG* (Figure 4A). Additionally, the enhancement of enzyme production was observed in the culture supernatants and cellular fractions by western blotting analysis (Figure 5B). As Hagihara et al. [20] previously reported that AmyK38 contains three Na^+^ ions in the protein structure, an NaCl solution was added to the growth medium to give the same amount of Na^+^ ions as resulting from the addition of 0.9% (w/v) Na_2_CO_3_. Additionally, we added an NH_3_ solution as a second alkaline solution. As a result, the production levels of AmyK38 significantly increased after addition of the NH_3_ solution, but no significant improvement of AmyK38 production was observed by addition of the NaCl solution (Additional file 2: Figure S2). These results strongly suggest that the optimal external pH for the production of AmyK38 is higher than the pH of standard growth medium used for culturing *B. subtilis*.

Under controlled pH conditions, the highest production level of AmyK38 (1.08 g l^-1^) was obtained using strain MGB874 (Figure 5A, 0.9% Na_2_CO_3_). To examine the influence of pH up-shift on the secretion stress response, we constructed strains R-1645 and R-1646 by introducing the *htrB-lacZ* gene fusion into strains 168 and MGB874, respectively. As shown in Table 1, the accumulations of β-galactosidase in strain 168 and MGB874 cells harboring pHYK38 were clearly reduced in the presence of Na_2_CO_3_, which resulted in an increase of the medium pH, in comparison with untreated cells (Table 1). Thus, the up-shift of external pH could alleviate the AmyK38-induced secretion stress response in *B. subtilis*, although a degree of the response remained.

**Table 1:** **Effect of pH up-shift on the secretion stress response in*B. subtilis***

**Strain**	**Genotype**	**Plasmid**	**Na_2_CO_3_^a^**	**LacZ activity^b^**
R-1645	168 Δ*amyE*::P_*htrB*_-*lacZ*	pHY300PLK	-	6.7 ± 0.3
R-1645	168 Δ*amyE*::P_*htrB*_-*lacZ*	pHY300PLK	+	6.5 ± 0.5
R-1646	MGB874 Δ*amyE*::P_*htrB*_-*lacZ*	pHY300PLK	-	9.5 ± 0.1
R-1646	MGB874 Δ*amyE*::P_*htrB*_-*lacZ*	pHY300PLK	+	9.1 ± 0.3
R-1645	168 Δ*amyE*::P_*htrB*_-*lacZ*	pHYK38	-	700 ± 86
R-1645	168 Δ*amyE*::P_*htrB*_-*lacZ*	pHYK38	+	200 ± 48
R-1646	MGB874 Δ*amyE*::P_*htrB*_-*lacZ*	pHYK38	-	1700 ± 36
R-1646	MGB874 Δ*amyE*::P_*htrB*_-*lacZ*	pHYK38	+	590 ± 85

^a^*B. subtilis* derivative strains were grown in 2xL-Mal medium at 30°C. At the transition phase (12 h), a Na_2_CO_3_ solution was added to the growth medium at a final concentration of 0.9% (w/v). -, No addition; +, Addition.

^b^LacZ activity was determined after 48 h of cultivation. β-galactosidase-specific activities are reported in units (U) calculated using the following formula: 1000 x A420/reaction time (min) x OD_600_ of culture.

To further improve AmyK38 production, we attempted to reduce the remaining secretion stress response by further increasing the external pH (Figure 5A, 1.2% Na_2_CO_3_ [w/v]). Increasing the external pH from pH 8.4 to 9.1 resulted in a decrease in the culture OD_600_ at 24 h of cultivation. Under this cultivation condition, the culture OD_600_ of strain MGB874 remained low until the end of the 72 h cultivation period, whereas increases in the culture OD_600_ of strain 168 were observed after only 24 h of cultivation. Thus, the severe growth inhibition of strain MGB874 cells would result in drastically decreased AmyK38 production (Figure 5A, 1.2% Na_2_CO_3_ [w/v]). Therefore, further enhancement of AmyK38 production is expected by the generation of a high alkaline-tolerant mutant of strain MGB874.

### **Reduced D-alanylation enhances production of AmyK38 in response to external pH up-shift**

Under normal growth conditions (pH 6.5), cell wall teichoic acids of *B. subtilis* contain ester-linked D-alanine residues, which reduce the density of negative charge in the cell wall [28]. Hyyrylainen et al. [18] reported that a D-alanylation-deficient strain (Δ*dltB*) displays increased α-amylase production compared to the wild-type strain [18]. Importantly, under alkaline conditions (pH 8.1), cell wall teichoic acids are non-alanylated due to the lability of D-alanine ester linkages at alkaline pH [18]. These observations suggest that the enhanced production of AmyK38 in response to an external pH up-shift might be due to the loss of D-alanine esters under alkaline conditions. Thus, we constructed D-alanylation-deficient mutants (Δ*dltB*) of strains 168 and MGB874, and then evaluated the production levels of AmyK38 in the presence and absence of added alkali.

As shown in Table 2, in the absence of Na_2_CO_3_, deletion of *dltB* enhanced AmyK38 production by strains 168 and MGB874. These data indicate that the absence of D-alanylation enhances the production levels of AmyK38 in both strains 168 and MGB874. Although AmyK38 does not require Ca^2+^ for activity, the absence of D-alanylation would increase the concentration of metal ions at the cell wall and membrane interface and metal ions could still influence its rate of folding. However, an external pH up-shift enhanced the production levels of AmyK38 even in the *dltB* mutants, and no significant differences in AmyK38 production were observed between the *dltB* mutants and their respective parental strains when the external pH was up-shifted (Table 2). Thus, the data presented here suggest that a deficit of D-alanylation is one of the factors enhancing production of AmyK38 under conditions of external pH up-shift.

**Table 2:** **AmyK38 production in*dltB*mutants with or without an up-shift of external pH**

**Strain**	**Plasmid**	**Na_2_CO_3_^a^**	**α-amylase activity(kU l^-1^)^b^**
168	pHYK38	-	2.9 ± 0.3
168ΔdltB	pHYK38	-	7.0 ± 0.3
MGB874	pHYK38	-	0.10 ± 0.01
874ΔdltB	pHYK38	-	0.27 ± 0.02
168	pHYK38	+	14.3 ± 0.2
168ΔdltB	pHYK38	+	14.8 ± 0.4
MGB874	pHYK38	+	20.8 ± 0.8
874ΔdltB	pHYK38	+	20.1 ± 0.2

^a^*B. subtilis* derivative strains were grown in 2xL-Mal medium at 30°C. At the transition phase (12 h), an Na_2_CO_3_ solution was added to the growth medium at a final concentration of 0.9% (w/v). -, No addition; +, Addition.

^b^α-amylase activity was determined after 72 h of cultivation.

In summary, the observed improvement of AmyK38 production by *B. subtilis* in response to an up-shift of external pH is considered to be a result of the following: (i) direct improvement of the folding efficiency and/or stability of AmyK38 prior to its release into the growth medium; (ii) indirect stabilization of AmyK38 through an increase in the net negative charge of the cell wall by not only the loss of protons, but also from the loss of D-alanine in the cell wall. (iii) reduced activities of the membrane-bound serine proteases HtrA and HtrB; and (iv) possible secondary effects of gene clusters activated or inactivated by increased external pH. These four possibilities cannot be discriminated by our present data, although the experiments using *dltB* mutants suggest that the loss of D-alanine esters due to an up-shift of external pH contributes to increased AmyK38 production. It should be mentioned that the alkaline α-amylase AmyK38 might fold effectively at alkaline external pH, which might increase the folding rate of AmyK38 as it emerges from the translocon, although experimental examination of this possibility is necessary.

## **Conclusions**

Although it is common knowledge that strict pH control in fermentations is critical for optimal enzyme yields, our results demonstrate for the first time that RocG is an important factor influencing enzyme production by *B. subtilis* through its role in preventing acidification of the growth medium. Higher external pH enables more efficient secretion of the alkaline α-amylase AmyK38, likely due to the reduced accumulation of unfolded AmyK38 at the membrane-cell wall interface, and thus lowering the secretion stress. As alkaline α-amylases would be expected to fold more effectively at alkaline pH, we will verify our findings using other non-alkaliphilic α-amylases, such as AmyL and AmyQ, in future experiments. In addition, our results suggested that the loss of D-alanine esters in high external pH conditions additionally contributes to increased AmyK38 production. Importantly, under controlled pH conditions, reduced-genome strain MGB874 was found to be a beneficial host for the production of AmyK38 and yielded the highest levels of AmyK38 reported to date. For further improvement of AmyK38 production, we are presently attempting to generate an alkaline-tolerant mutant to reduce the remaining secretion stress response in *B. subtilis*.

## **Methods**

### **Bacterial strains, plasmids, and growth media**

The bacterial strains and plasmids used in this study are listed in Table 3. *E. coli* HB101 (Takara Bio, Inc.) was used as the host for plasmid preparation and was grown in Luria-Bertani (LB) medium (1% [w/v] Bacto tryptone [Difco], 0.5% [w/v] Bacto yeast extract [Difco], and 1% [w/v] NaCl). For the preparation of *B. subtilis* competent cells, Spizizen minimal medium [29] was used as the basal medium. LB agar medium containing 1.5% (w/v) agar and either 100 μg ml^-1^ ampicillin (Amp), 100 μg ml^-1^ spectinomycin (Sp), 10 μg ml^-1^ chloramphenicol (Cm), or 20 μg ml^-1^ neomycin (Nm), as appropriate, was used as the selective medium. The protoplast transformation method [30] was used to introduce plasmids into *B. subtilis*, and positive transformants were selected on DM3 medium [30] containing 45 μg ml^-1^ tetracycline (Tet). For enzyme production, 2xL-Mal medium (2% [w/v] tryptone [Difco], 1% [w/v] yeast extract [Difco], 1% [w/v] NaCl, 7.5% [w/v] maltose monohydrate, 7.5 μg ml^-1^ manganese sulfate 4–5 hydrate, and 15 μg ml^-1^ Tet) with an initial pH of 6.8 was used. As necessary, IPTG was added to a final concentration of 1 mM, and various concentrations of Na_2_CO_3,_ NaCl, and NH_3_ solutions were added after 12 h of cultivation.

**Table 3:** Bacterial strains and plasmids used or constructed in this study

**Strain or plasmid**	**Relevant properties^†^**	**Source or reference**
Strain		
*Bacillus subtilis*		
168	*trpC2*	[5]
MGB625	MGB604 Δ(*yeeK-yesX*)	[8]
MGB723	MGB625 Δ(*pdp-rocR*)	[8]
MGB874	MGB860 Δ(*yncM-yndN*)	[8]
MGB625Δr1	MGB625 Δ(*pdp-yxeD)*::*cat*	[11]
MGB625Δr2	MGB625 Δ(*yxeC-yxnA*)::*cat*	[11]
MGB625Δr3	MGB625 Δ(*yxaD-fbp*)::*cat*	[11]
MGB625Δr4	MGB625 Δ(*yydD-rocR*)::*cat*	[11]
MGB625ΔyydD-yycN	MGB625 Δ(*yydD-yycN*)::*cat*	[11]
MGB625ΔrapG-phrG	MGB625 Δ(*rapG-phrG*)::*cat*	[11]
MGB625ΔrocF-rocR	MGB625 Δ(*rocF-rocR*)::*cat*	[11]
MGB625Δpdp-rocD	MGB625 Δ(*pdp-rocD*)::*cat*	This study
874DEFR	MGB874 *cat-rocDEF-rocR* was inserted at the Δ(*pdp-rocR*) locus	[11]
874RocR	MGB874 *cat -rocR* was inserted at the Δ(*pdp-rocR*) locus	This study
874DEFRΔrocG	874DEFR Δ*rocG*::*spec*	[11]
874DEFRΔrocABC	874Δ DEFRΔ*rocABC*::*spec*	This study
R-726	MGB874 Δ*aprE*::P_spac_-*rocG spec* (pAPNC213-rocG)	This study
R-1645	168 Δ*amyE*::P_htrB_-*lacZ cat* (pDN110)	This study
R-1646	MGB874 Δ*amyE*::P_htrB_-*lacZ cat* (pDN110)	This study
R-1926	MGB874 Δ*aprE*::P_spac_-*rocG spec* Δ*amyE*::P_htrB_-*lacZ cat* (pDN110)	This study
168ΔcssRS	168 Δ*cssRS*::*spec*	This study
874ΔcssRS	MGB874 Δ*cssRS*::*spec*	This study
168ΔdltB	*trpC2* Δ*dltB*::*neo*	This study
874ΔdltB	MGB874 Δ*dltB*::*neo*	This study
*Escherichia coli*		
HB101	*supE44 Δ(mcrC-mrr) recA13 ara-14 proA2 lacY1 galK2 rpsL20 xyl-5 mtl-1 leuB6 thi-1*	Takara Bio
Plasmid		
pHY300PLK	Shuttle vector for *E. coli* and *B. subtilis*	Takara Bio
pHYK38	pHY300PLK carrying the gene for AmyK38 from *Bacillus* sp. strain KSM-K38 fused to the promoter, SD, and leader peptide sequences of *egl-237*, containing *amp* and *tet*	This study
pAPNC213	The integration vector containing P_spac_ promoter into the *aprE* locus by double crossover using *spec*	[35]
pAPNC213-rocG	pAPNC213 containing the SD sequence and coding region of *rocG* (−25 to 1275, 1325 bp)	This study
pDL2	Integration vector for introduction of single-copy transcriptional fusions to *lacZ* by double crossover at the *amyE* locus (*cat*)	[36]

^†^Antibiotic resistance genes are abbreviated in the table as follows: *amp*, ampicillin; *tet*, tetracycline; *cat*, chloramphenicol; *spec*, spectinomycin; and *neo*, neomycin*.* SD, Shine-Dalgarno.

### **Construction of*B. subtilis*mutant strains**

Primers used for the construction of *B. subtilis* mutants and plasmids are listed in (Additional file 3: Table S1). DNA manipulation techniques were as described previously [31]. *B. subtilis* strain 874DEFRΔrocABC, in which the *rocABC* operon was substituted with the Sp-resistance gene (*spec*), was constructed from strain 874DEFR as follows. The *spec* gene was first amplified from pDG1727 [32] using primers Spf and Spr. The approximately 1.0-kb flanking sequences located upstream (UProcABC) and downstream (DNrocABC) of the *rocABC* operon were then amplified by PCR from the strain 874DEFR genome using the primer pairs rocABCFW and rocABC/SpR, and rocABC/SpF and rocABCRV, respectively. The UProcABC and DNrocABC fragments overlapped with *spec* by 20 bp at the downstream and upstream ends, respectively. The three PCR fragments were then ligated in the order UProcABC-spec-DNrocABC by gene splicing using overlap extension PCR (SOE-PCR) [33] and the primers rocABCFW2 and rocABCRV2. The resulting 3.2-kb PCR product was used for the transformation of strain MGB874, and a positive Sp-resistant transformant was selected and designated as strain 874DEFRΔrocABC. Deletion of the *rocABC* operon was confirmed by PCR using primers rocABCFW and rocABCRV. A similar approach was used for the construction of mutant strains MGB625Δpdp-rocD, 168ΔcssRS, 874ΔcssRS, 168ΔdltB, and 874ΔdltB using the primers listed in (Additional file 3: Table S1). A 0.9-kb Cm-resistance gene (*cat*) fragment and 0.8-kb Nm-resistance gene (*neo*) fragment were PCR amplified from plasmids pC194 [34] and pUB110 [33], respectively.

*B. subtilis* strain 874rocR was constructed using a similar approach to that used for the construction of strain 874DEFR [11]. Briefly, strain MGB874 was transformed with chromosomal DNA from strain MGB625Δpdp-rocD, which only contains the *rocRcat* gene in the *pdprocR* region of strain MGB625. As a result of the transformation, the *rocR* and *cat* fragment were reintroduced into the Δ*pdprocR* region in strain MGB874.

*B. subtilis* strain R-726, in which the *rocG* gene was expressed under control of the IPTG-inducible P_spac_ promoter from pAPNC213, was constructed as follows. A *rocG* gene fragment containing the ribosome-binding site of *rocG* was amplified and then cloned into the SalI/SacI sites of pAPNC213 [35]. The resulting plasmid, named pAPNC213-rocG, was transformed into strain MGB874 to integrate the *rocG* gene into the *aprE* site by homologous recombination. A Sp-resistant transformant was selected and designated as strain R-726.

A transcriptional *htrBlacZ* fusion gene was generated by modification of plasmid pDL2 [36]. First, plasmid pDN110 was constructed by cloning a 263-bp PCR-generated fragment (synthesized with primers YVTAPF and YVTAPR) using the procedure described by Noone et al [37]. Strains R-1645, R-1646, and R-1926 were generated from strains 168, MGB874, and R-726, respectively, by the introduction of plasmid pDN110 and selection of Cm-resistant transformants.

### **Construction of expression vectors for the evaluation of exogenous enzyme production**

Plasmid pHYK38 was used for the expression of alkaline α-amylase AmyK38 from *Bacillus* sp. strain KSM-K38 [12] and was constructed as follows. A 1.5-kb DNA fragment coding for the mature form of AmyK38 was PCR amplified using genomic DNA extracted from *Bacillus sp.* KSM-K38 strain as template and primers K38matu-F2 (ALAA) and K38matu-R (XbaI) (Additional file 3: Table S1). In addition, a 0.6-kb DNA fragment including the promoter, Shine-Dalgarno (SD) sequence, and coding region for the signal sequence of the alkaline cellulase Egl-237 was PCR amplified using *Bacillus* sp. KSM-S237 genome as template and primers S237ppp-F2 (BamHI) and S237ppp-R2 (ALAA) (Additional file 3: Table S1). The amplified 1.5-kb and 0.6-kb fragments were fused together by SOE-PCR with primers S237ppp-F2 (BamHI) and SP64K38-R (XbaI) (Additional file 3: Table S1) to generate a 2.1-kb DNA fragment containing the AmyK38 gene downstream of the promoter, SD sequence, and coding region of the *egl-237* signal sequence. The 2.1-kb DNA fragment was then inserted into the BamHI-XbaI cleavage site of the shuttle vector pHY300PLK (Takara Bio, Inc.) [38].

### **Viable cell counts**

The detection of viable bacteria was performed by cultivation on agar plates and enumeration of colony-forming units (CFU). For each bacterial sample, a dilution series of cell suspensions was plated on LB agar plates, incubated overnight at 37°C, and colonies formed on the agar plates were then enumerated.

### **Culture method for assessment of AmyK38 production**

*B. subtilis* mutants were transformed with pHYK38 by the protoplast transformation method, as previously described [30]. To assess AmyK38 production levels, cells were pre-cultured in LB medium supplemented with 15 μg ml^-1^ Tet at 30°C for 15 h with shaking at 120 rpm, and 600 μl of the pre-culture broth was then inoculated into 30 ml 2xL-Mal medium in a 500-ml Sakaguchi flask. After further cultivation at 30°C with shaking at 120 rpm, cells were removed by centrifugation at 9,000 x *g* for 10 min, and α-amylase activity in the culture supernatant was then determined, as described below.

### **Amylase activity assay**

α-amylase activity in culture supernatants was measured using a Liquitec Amy EPS kit (Roche Diagnostics), which uses 4,6-ethylidene-4-nitrophenyl-α-D-maltoheptaoside as a substrate [39]. Culture broth supernatants were diluted 676-fold with 125 mM glycine-NaOH buffer (pH 10.0). As the pH of diluted samples was 10.0 after dilution, it appears that the pH of the growth medium did not influence the measured α-amylase activity. After sample preparation, 100 μl of R1-R2 mixture (R1 [coupling enzyme]: R2 [amylase substrate] = 5 : 1 [vol] was mixed with 50 μl of diluted sample solution, and the amount of *p*-nitrophenol released during incubation at 30°C was quantitatively determined by the change in absorbance at 405 nm (*A*_405_). The amount of enzyme required for the release of 1 μmol *p*-nitrophenol per minute was defined as 1 unit (U).

### **Purification of AmyK38**

For the purification of AmyK38, *B. subtilis* strain 168 harboring pHYK38 was cultivated in 2xL-Mal medium at 30°C for 72 h. The purification procedure was performed as described by Hagihara et al. [12], with only minor modifications. Briefly, the centrifuged supernatant of the culture broth was loaded onto a Biogel P-6 DG desalting column (Bio-Rad Laboratories) equilibrated with 10 mM Tris–HCl buffer (pH 7.0). The eluate was collected as a single fraction and subjected to further purification by chromatography using a DEAE-Toyopearl 650 M column (Tosoh, Tokyo, Japan) and the identical protocol. Purified protein was quantified by a modified method of Lowry et al. [40] using the DC Protein Assay kit (Bio-Rad) and bovine serum albumin as the standard. The specific activity of purified AmyK38 towards 4,6-ethylidene-4-nitrophenyl-α-D-maltoheptaoside, as measured using a Liquitec Amy EPS kit (Roche Diagnostics) was 22,800 U mg protein^-1^.

### **Antibodies**

Rabbit polyclonal antibodies against purified AmyK38 protein were prepared by Operon Biotechnology Co. (Tokyo, Japan).

### **Western blotting**

Culture samples were harvested, and cells were then separated from the culture medium by centrifugation. The resulting supernatants were diluted 40-fold with 10 mM Tris–HCl (pH 7.0) containing Complete Mini Protease Inhibitor Cocktail Mixture (Roche Applied Science). Cells were washed twice with fresh 2xL-Mal medium, and re-suspended to an OD_600_ of 3.0 in 10 mM Tris–HCl (pH 7.0) containing the cocktail mixture. After adding 3x SDS-PAGE Loading Buffer (BioVision), the samples were incubated at 95°C for 1 min. For Western blotting analysis, the samples were subjected to sodium dodecyl sulfate polyacrylamide gel electrophoresis (SDS-PAGE) using 10% polyacrylamide gels (Ready Gels; Bio-Rad Laboratories) and 1x Tris-glycine-SDS running buffer (Bio-Rad Laboratories). The separated proteins were transferred to a PVDF membrane using a semi-dry transfer apparatus (Bio-Rad Laboratories) and then probed with primary antibodies against AmyK38 (1:2500) and visualized with a Western Breeze Colorimetric kit (Invitrogen) according to the manufacturer’s instructions. MagicMark XP Western Protein Standard (Invitrogen) was used as a protein standard.

### **Preparation of culture supernatants for assessment of AmyK38 stability**

*B. subtilis* cells harboring pHYK38 were grown for 48 h in 30 ml 2xL-Mal medium, and cells were then removed by centrifugation at 9,000 x *g* for 30 min at 4°C. The obtained supernatants were filtered through a 0.22-μm pore-size MF microfilter (Millipore) to ensure the complete removal of cellular material. The cell-free supernatants were incubated at 30°C, and samples were removed at various intervals for the determination of α-amylase activity and Western blotting analysis.

### **β-galactosidase assay**

The β-galactosidase assay was performed according to the method described by Hyyryläinen et al. [15]. Briefly, cells were collected from 50 μl samples by centrifugation at 12,000 x *g* for 2 min at 4°C and were then washed once in 0.5 ml ice-cold 25 mM Tris–HCl (pH 7.4). After centrifugation at 12,000 x *g* for 2 min at 4°C, the pellet was first re-suspended in 0.64 ml Z buffer (60 mM Na_2_HPO_4_-H_2_O, 40 mM NaH_2_PO_4_-H_2_O, 10 mM KCl, and 1 mM MgSO_4_-7H_2_O) supplemented with 2 mM dithiothreitol (DTT) and then mixed with 0.16 ml lysozyme solution (2.5 mg/ml). After a 10-min incubation at 37°C, the sample was mixed with 8 μl 10% (v/v) Triton-X 100 and then stored at −30°C until assayed. The assay was initiated by the addition of 0.2 ml *ο*-nitrophenyl-β-D-galactoside (ONPG; 4 mg dissolved in Z buffer) to the pre-warmed sample, followed by incubation of the resulting mixture at 30°C. The reaction was terminated once the mixture turned yellow by adding 0.4 ml 1 M Na_2_CO_3_, and the total incubation time was recorded. β-galactosidase-specific activities are reported in units (U) and were calculated using the formula: 1000 x *A*_420_/reaction time (min) x OD_600_ of culture.

### **Measurement of ammonia concentration**

The concentration of ammonia in culture supernatants was determined by enzymatic analysis according to the F-Kit UV method (Boehringer GmbH).

### **Measurement of sugar concentration**

Sugars were analyzed by the phenol-sulfuric acid method [41]. Briefly, 0.2-ml aliquots of diluted culture supernatants were mixed with an equal volume of 5% (w/v) phenol. One milliliter of concentrated sulfuric acid was then added and the mixture was mixed well. Following incubation at room temperature for 30 min, the absorbance of each sample was measured at 492 nm (*A*_492_). The concentration of sugars was calculated with a standard curve using known concentrations of glucose.

### **Quantitative real-time PCR**

Total RNA was extracted from *B. subtilis* cells, as described previously [42], and reverse transcribed to cDNA using an AffinityScript QPCR cDNA Synthesis kit (Stratagene). As negative controls, all RNA samples were subjected to the identical reaction conditions without reverse transcriptase. qRT-PCR amplification, detection, and analysis were performed with the Mx3005P Real-time PCR System (Stratagene) and Brilliant II Fast SYBR Green QPCR Master Mix (Stratagene). The primer sequences used for qRT-PCR were designed using Primer 3 (version 0.4.0) [43] and are listed in (Additional file 4: Table S2). The source code of Primer 3 is available at http://frodo.wi.mit.edu/primer3/.

qRT-PCR was performed in 25-μl reaction mixtures consisting of 1xSYBR Green master mix, 0.4 μM of each forward and reverse primer, 3.2 μM reference dye, and 10 μl template. The PCR conditions were 95°C for 2 min, followed by 40 cycles of 95°C for 5 s and 60°C for 20 s. Amplified products were confirmed by dissociation curve analysis. To estimate the quantity of initial template DNA in samples, serial qRT-PCRs were performed by amplifying inserted target DNA in pUC118 (Takara Bio, Inc.) with specific primers. For each target gene, a standard curve was generated using the log of the quantity of initial template DNA plotted against the threshold cycle (Ct) values for the standard wells. The generated standard curves were used to convert the Ct values of each amplified gene in the cDNA preparations to copy numbers of cDNA molecules. The estimated copy number was normalized to the value for 16 S rRNA, as previously described [44].

## **Abbreviations**

CFU, Colony-forming units; CssRS, Control secretion stress Regulator and Sensor; GRAS, Generally Regarded as Safe; IPTG, Isopropyl-β-D-thiogalactopyranoside; qRT-PCR, Quantitative real-time PCR; SDS-PAGE, Sodium dodecyl sulfate polyacrylamide gel electrophoresis; Amp, Ampicillin; Sp, Spectinomycin; Cm, Chloramphenicol; Nm, Neomycin; Tet, Tetracycline.

## **Competing interests**

The content of this manuscript is relevant to a patent application made by Kao Corporation (Patent no. JP2010-187588A); however, all authors declare that they have no competing interests.

## **Authors' contributions**

NO, KO, and KA initiated and coordinated the project. KM and YK drafted the manuscript, constructed mutant strains, evaluated production levels of AmyK38, and measured pH and ammonium concentrations in the growth medium. KM performed qPCR, Western blotting, and β-galactosidase assays. MT constructed the expression vector for AmyK38 and developed the α-amylase activity assay. NO and KO supervised the study and reviewed results. All authors read and approved the final version of the manuscript.

## Supplementary Material

Additional file 1 **Figure S1.** Determination of the gene deletions responsible for decreased AmyK38 production. *B. subtilis* strains 168, MGB625, MGB723, MGB874, and MGB625 derivative strains harboring pHYK38 were cultured in 2xL-Mal medium with shaking at 30°C. α-amylase activities in the growth media were measured after 72 h cultivation. All results presented are the averages of three individual experiments. Error bars represent standard deviations (n=3). Please refer to [11] concerning the construction of the MGB625 derivative strains. Click here for file

Additional file 2 **Figure S2.** Effect of NaCl and NH_3_ addition on AmyK38 production. *B. subtilis* strains 168 and MGB874 harboring pHYK38 were grown in 2xL-Mal medium at 30°C. At the transition phase (12 h), an NaCl or NH_3_ solution was added to the growth medium at final concentrations of 1.0% (w/v) or 0.23% (v/v), respectively. Culture OD_600_ (open circles) and external pH (open triangles) were measured after 24 h of cultivation, and α-amylase activities (black bars) were measured after 72 h cultivation. All results presented are the averages of three individual experiments. Error bars represent standard deviations (n=3). Click here for file

Additional file 3 **Table S1.** Primers used for the construction of mutants and plasmids. Click here for file

Additional file 4 **Table S2.** Primers used in the real-time PCR analysis. Click here for file
