# Supplementary figures and images for: High external pH enables more efficient secretion of alkaline α-amylase AmyK38 by Bacillus subtilis
Source: Microb Cell Fact. 2012 Jun 8;11:74. doi: 10.1186/1475-2859-11-74 (PMC3424145; doi:10.1186/1475-2859-11-74)

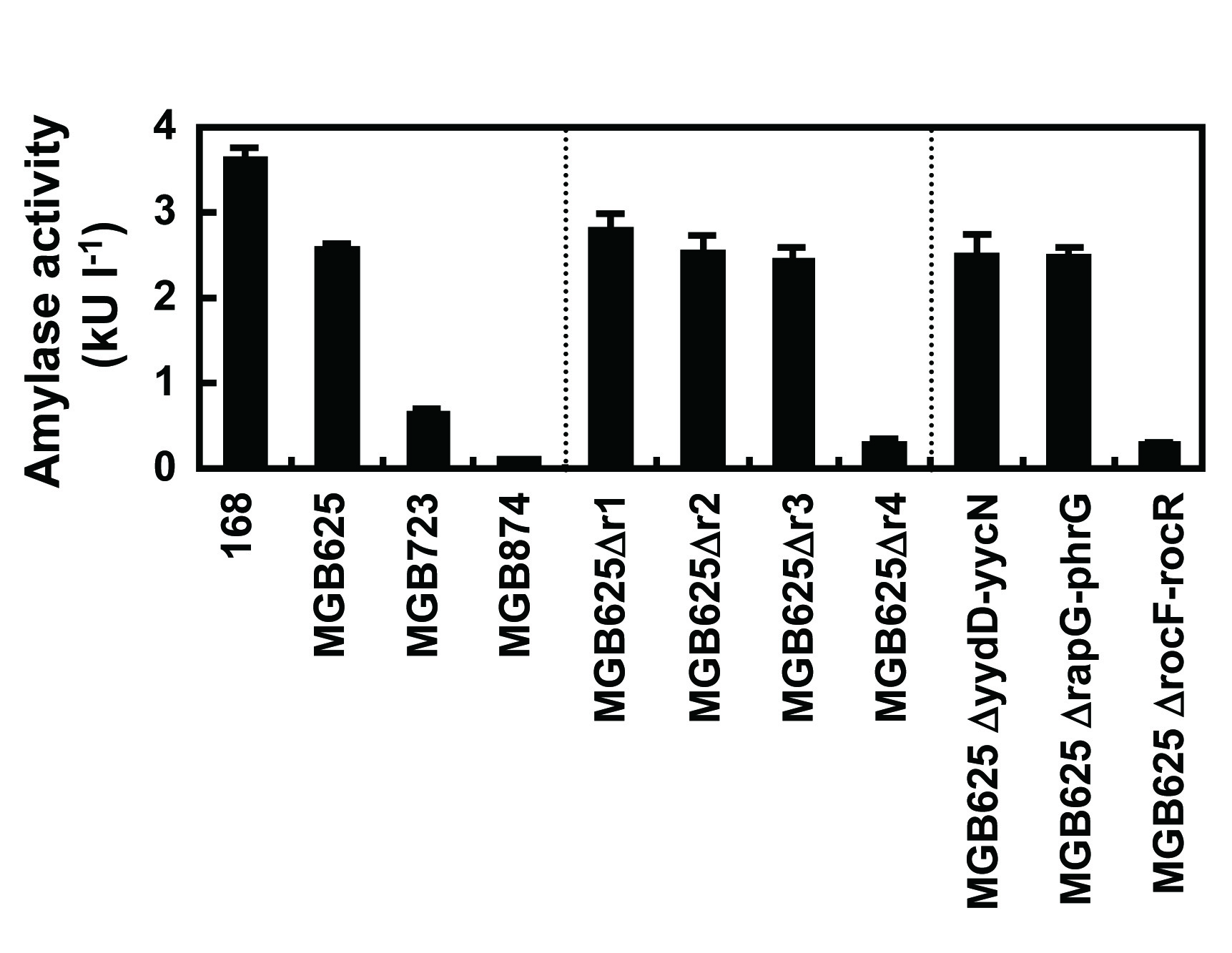

Supplement: Additional file 1 — Figure S1. Determination of the gene deletions responsible for decreased AmyK38 production. B. subtilis strains 168, MGB625, MGB723, MGB874, and MGB625 derivative strains harboring pHYK38 were cultured in 2xL-Mal medium with shaking at 30°C. α-amylase activities in the growth media were measured after 72 h cultivation. All results presented are the averages of three individual experiments. Error bars represent standard deviations (n=3). Please refer to [11] concerning the construction of the MGB625 derivative strains. [file 1475-2859-11-74-S1.jpeg]

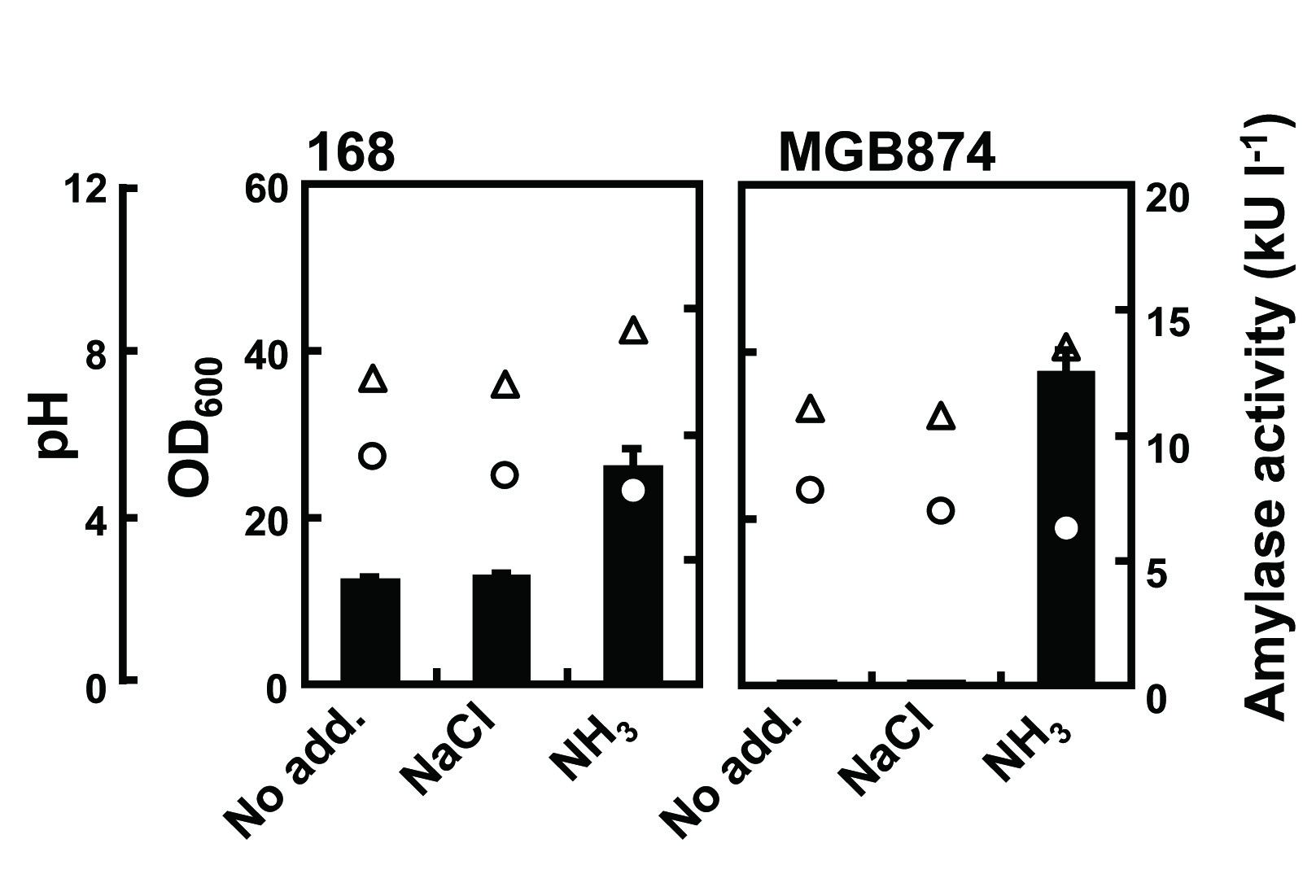

Supplement: Additional file 2 — Figure S2. Effect of NaCl and NH3 addition on AmyK38 production. B. subtilis strains 168 and MGB874 harboring pHYK38 were grown in 2xL-Mal medium at 30°C. At the transition phase (12 h), an NaCl or NH3 solution was added to the growth medium at final concentrations of 1.0% (w/v) or 0.23% (v/v), respectively. Culture OD600 (open circles) and external pH (open triangles) were measured after 24 h of cultivation, and α-amylase activities (black bars) were measured after 72 h cultivation. All results presented are the averages of three individual experiments. Error bars represent standard deviations (n=3). [file 1475-2859-11-74-S2.jpeg]
