# Supplementary material for: High external pH enables more efficient secretion of alkaline α-amylase AmyK38 by Bacillus subtilis
Source: Microb Cell Fact. 2012 Jun 8;11:74. doi: 10.1186/1475-2859-11-74 (PMC3424145; doi:10.1186/1475-2859-11-74)
Supplement: Additional file 3 — Table S1. Primers used for the construction of mutants and plasmids. [file 1475-2859-11-74-S3.doc]

**Additional file 3: Table S1** Primers used for the construction of mutants and plasmids.

| Primer | Sequence (5'-3')^a^ | Purpose in this study^b^ |
| --- | --- | --- |
| Spf | ATCGATTTTCGTTCGTG | Cloning of *spec* |
| Spr | CATATGCAAGGGTTTATTG |
| CmFW | CAACTAAAGCACCCATTAG | Cloning of *cat* |
| CmRV | CTTCAACTAACGGGGCAG |
| Nmf | GGGAATGAGTTTATAAAATAAAAA | Cloning of *neo* |
| Nmr | CCTTTATTCCGTTAATGCGC |
| rocABCFW | ATGATAAATAAGCCCGCAGC | Construction of  874DEFRΔrocABC |
| rocABC/SpR | CAATAAACCCTTGCATATGAAAAAGCTCTCCGGGAGGCC |
| rocABC/SpF | CACGAACGAAAATCGATATGTAGTCCCCCTCGTGTTA |
| rocABCRV | ATGGATGAGTACAGCCGGCT |
| rocABCFW2 | ACATACCGTAAAAACCAATC |
| rocABCRV2 | GAGGATCGCAAGGACGGGAA |
| pdpFW | TCCAGCGTAAAGATTGTCACC | Construction of  MGB625Δpdp-rocD |
| pdp/CmR | CTAATGGGTGCTTTAGTTGGCTGATTCATACGTTAATTACAG |
| rocR/CmF | CTGCCCCGTTAGTTGAAGTTGTTGGCTCCGTAATGAGAC |
| rocRRV | GATTTTCCGTTCTTGAAGGGC |
| pdpFW2 | CATTTTCAGTTAAGTTATTTGCGG |
| rocRRV2 | TTGGGTCTTCATTCATCGTGG |
| cssRSFW | GCGCAGCTGTGAAGAGTCAC |  |
| cssRS/SpR | CACGAACGAAAATCGATGGCTCTTCACATCCTTTCAA |
| cssRS/SpF | CAATAAACCCTTGCATATGACTGTAGATGTTTTGCAGTC |
| cssRSRV | CGTCCGTGCGTAAGAGGTCC |
| cssRSFW2 | AAATCAGTAATGGTATCGCT |
| cssRSRV2 | CAGACATGCCTTTTACCATA |
| dltB FW | TGCCTTCAATCAGGCGG | Construction of  Δ*dltB* mutants |
| dltB/NmR | TTTTTATTTTATAAACTCATTCCCATGTTGTATGCTTGAAATC |
| dltB RV | TCGTTGAATAATACAGATC |
| dltB/NmF | GCGCATTAACGGAATAAAGGATTCTCGCTATTGTGATC |
| dltB FW2 | TTGAAGAGCTGAAGAAGT |
| dltB RV2 | TCACTTTCCACGTCTGA |
| RocG.F.SalI | AAAAgtcgacTTACATTACAGCCGGCCAAAAAAAC | Cloning of *rocG* |
| RocG.R.SacI | AAAAgagctcTCATTAGACCCATCCGCGGAAACGC |
| YVTAPF | TACACggatccAACGGTTATTCATTTATCGTTACATATTC | Cloning of *htrB* control region |
| YVTAPR | GTGTAgaattcGGCTCTTCACATCCTTTCAAC |
| S237ppp-F2(BamHI) | CCCGGATCCAACAGGCTTATATTTA | Construction of pHYK38 |
| S237ppp- R2 (ALAA) | TTCAATCCATCTGCTGCAAGAGCTGCCGG |
| K38matu-F2 (ALAA) | GCTCTTGCAGCAGATGGATTGAACGGTACG |
| K38matu-R (XbaI) | TTGGTCTAGACCCCAAGCTTCAAAGTCGTA |
| ^a^ Overlapping sequences for SOE-PCR are underlined and restriction enzyme recognition sites are indicated in lowercase letters. ^b^*cat*, chloramphenicol; *spec*, spectinomycin; *neo*, neomycin. | | |
