# Supplementary material for: High external pH enables more efficient secretion of alkaline α-amylase AmyK38 by Bacillus subtilis
Source: Microb Cell Fact. 2012 Jun 8;11:74. doi: 10.1186/1475-2859-11-74 (PMC3424145; doi:10.1186/1475-2859-11-74)
Supplement: Additional file 4 — Table S2. Primers used in the real-time PCR analysis. [file 1475-2859-11-74-S4.doc]

Additional file 4: Table S2**. Primers used in the real-time PCR analysis.**

| Target gene | Forward primer (5'-3') | Reverse primer (5'-3') |
| --- | --- | --- |
| 16Sr RNA | TCCGCAATGGACGAAAGTCT | ACGATCCGAAAACCTTCATCA |
| *amyK38* | CATCCGATGCATGCAGTTAC | GGTTCACGTACACGGATACA |
| *htrA* | GCAGCAAACGCAATCTGTTA | CTGATTTGGAGGACGGCTAA |
| *htrB* | ACCGGACAATTGATGTGGAT | CACCTTTGCCCAATTGATCT |
